# Maximum MLC opening effect in dynamic delivery of IMRT: leaf‐positional analysis

**DOI:** 10.1120/jacmp.v6i2.2076

**Published:** 2005-05-21

**Authors:** Piotr Zygmanski, Fred Hacker, Scott Friesen, Robin Rodenbush, Hsiao‐Ming Lu, Lee Chin

**Affiliations:** ^1^ Department of Radiation Oncology Brigham and Women's Hospital and Harvard Medical School Massachusetts General Hospital and Harvard Medical School Boston Massachusetts 02114 U.S.A.; ^2^ Department of Radiation Oncology Massachusetts General Hospital and Harvard Medical School Boston Massachusetts 02114 U.S.A.

**Keywords:** IMRT delivery, quality assurance

## Abstract

The analysis of dynamic multileaf collimator (MLC) positions for the delivered intensity‐modulated radiotherapy (IMRT) plans is crucial in that it may capture dose delivery problems otherwise difficult to observe and quantify in the conventional dosimetric measurements with film or with an ionization chamber. In some IMRT systems, delivery of IMRT fields starts with a maximum MLC opening (roughly the shape of the target in the beam's‐eye view) and then proceeds to the subsequent dynamic MLC subfields. No irradiation is required in going from the initial segment (maximum opening) to the next one, and theoretically, no dose should be delivered in that initial moment. However, due to a finite sampling time of the MLC controller, the finite speed of the MLC, and a finite leaf tolerance, there may be some dose delivered between the first and the second segment. The amount of the excess dose is higher for larger dose rates and for a smaller number of the total monitor units per IMRT field. The magnitude of the dose errors could be in the order of a few percent. Effects similar to the maximum MLC opening may occur in other situations as well, for instance, when leaves are forced to move over large distances in a short time. Confounding this are dose errors due to the uncertainty in the MLC transmission. The analysis of the actual leaf positions recorded in the dynamic MLC log file is helpful in differentiating between the two types of errors and in determining the optimal dynamic MLC delivery parameters.

PACS numbers: 87.53.‐j, 87.90.+y

## I. INTRODUCTION

Leaf‐positional analysis in intensity‐modulated radiotherapy quality assurance (IMRT QA) has been reported in the literature^(1–8)^; nonetheless, it has not yet become a widely used routine procedure for patient‐specific IMRT QA. There are two ways of obtaining the actual leaf positions for a delivered IMRT field: either by a direct measurement with a radiographic imaging system^(6^ ^–^ ^8)^ or by saving the multileaf collimator (MLC) controller log files.^(3^ ^–^ ^5)^ The latter method is not capable of detecting MLC miscalibrations/offsets, disturbance of leaf motion by gravitation, and similar effects.^(9)^ But it can definitely be used for the analysis of the dynamic MLC effects related to dose rate *R*, total number of monitor units ${\text{MU}}_{\text{tot}}$, MLC tolerance TOL, and maximum MLC speed $v_{\text{max}}$, as well as for understanding other MLC control issues, such as the nature of sampling of the leaf trajectory,^(10)^ since a log file represents a recording of the MLC controller's activity.

In this communication, we report a potential problem when the MLC is run in a dynamic MLC (dMLC) mode, especially when the motion starts from the maximum MLC opening, which is roughly the target shape in the beam's‐eye view (BEV). Delivering IMRT fields at high dose rates *R* or forcing the leaves to move over large distances in a short time during other stages of MLC motion aggravates the problem. A similar problem, commonly referred to as the overshoot phenomenon, has been reported for the step‐and‐shoot delivery.^(11)^ The maximum MLC opening effect can be most clearly seen in leaf‐positional analysis based on the MLC log file data.

**Figure 1:**
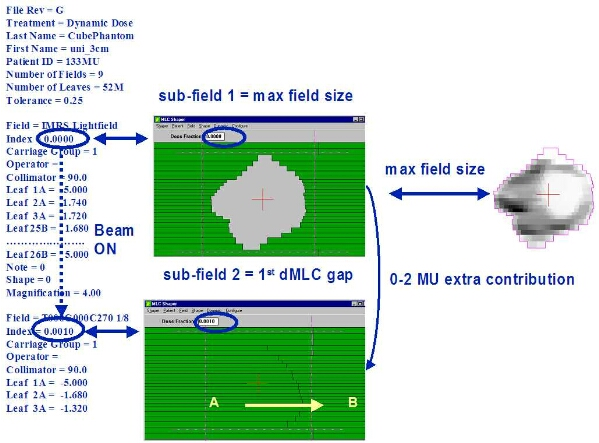
Schematic chart showing the initial content of the dMLC ASCII file (left) and the first two subfields using the Shaper™ (Varian). The MLC starts with the maximum opening, then closes and continues to the subsequent small‐gap subfield. Arrows indicate direction of motion.

The maximum MLC opening corresponding to a given dMLC fluence pattern is shown in Fig. 1. For brevity of expression, we use “maxMLC” to denote the maximum MLC opening. It is possible that the first MLC subfield is specified in a dMLC file as a subfield for zero dose fraction (“Index” in the MLC file). Ideally, there should be no dose delivered between the first subfield (maxMLC) and the second subfield (regular dMLC subfield, or segment), or at least the dose should be negligible. However, if the dose fraction for the second subfield is nonzero (see the content of the ASCII MLC file on the left in Fig. 1), the delivery is going to be performed in dynamic MLC mode with the beam ON. This behavior is consistent with how the dynamic MLC was designed: beam OFF when two subsequent subfields have the same dose fraction and beam ON when the dose fraction is incremented. Typically, during this initial time span, the leaves are supposed to move over the distances of several centimeters (the width of the target in BEV). This is not possible due to the mechanical constraint imposed by the maximum MLC velocity ($v_{\text{max}}$ is typically a few centimeters per second). The MLC controller should spot this problem and stop the beam, then correct the leaf positions and turn the beam ON again. However, the controller may not always intervene as indicated in an example in Fig. 1; it fails to do so before the first one or two sampling cycles elapse. The reason for the MLC controller failure is the combined effect of the finite sampling time Δt (Δt≈50 ms), the finite MLC tolerance TOL (TOL≈2 mm in practice), and the high inertia of the mechanical parts.

**Figure 2:**
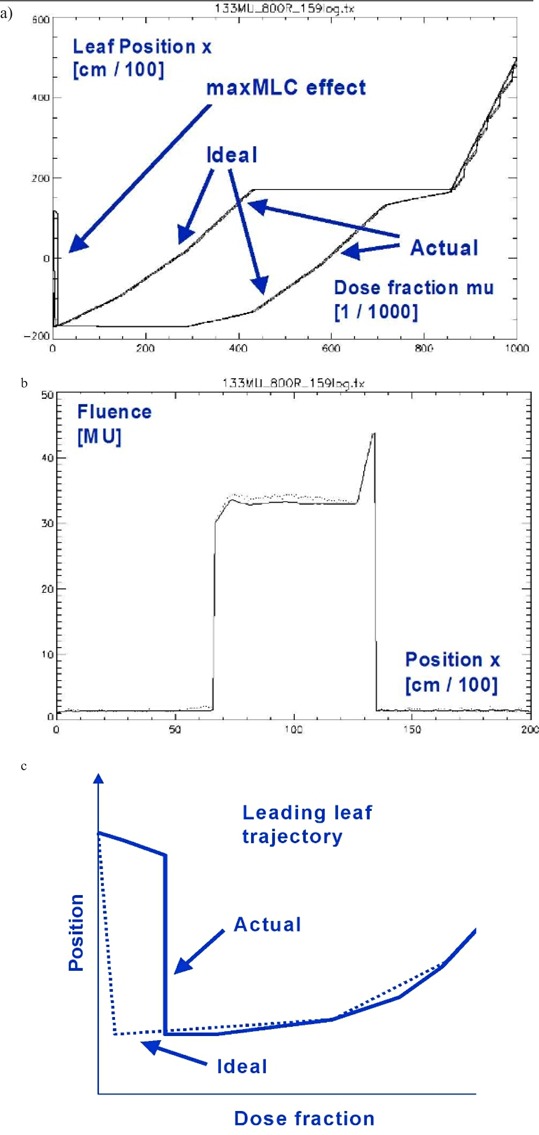
Example of log file analysis for a selected leaf pair. (a) Leaf trajectories (ideal and actual) for a leading and following leaf as a function of monitor unit fraction mu ∈ [0,1]. The actual is noisier. (b) The resulting fluence profile. Wiggles are due to local deviations from the ideal trajectory. The maxMLC effect is seen as the delayed trajectory of the leading leaf. (c) A schematic representation of the ideal and actual trajectories for the leading leaf in the beginning of the travel.

An example of leaf trajectories for a leaf pair with the presence of maxMLC effect and the corresponding dose errors are shown in Fig. 2. The IMRT field was delivered with ${\text{MU}}_{\text{tot}}=133\;\text{MU}$ and R=800 MU/min. In general, about a half or two‐thirds of the planning tumor volume (PTV) in the BEV receives an extra dose from the maximum MLC opening subfield for such a high dose rate, for example, using the Novalis™ (BrainLAB) and MULTI‐ACCESS™ (IMPAC) systems. The overall effect from all IMRT fields on a composite dose to PTV is more or less to uniformly increase the dose. This can be explained as follows. Initially, the MLC is opened showing the maximum MLC opening for a given fluence. In order for the MLC to go to the first real subfield, the MLC has to close. This is achieved by moving the leading leaf bank leaves (A) backward (against the direction the leaves are traveling in dynamic delivery, from left to right in Fig. 1). During MLC closing, the beam is ON because of the limitations of the inherent Novalis/IMPAC configuration. Therefore, some extra dose is deposited when the MLC is half or one‐third closed, thus always causing irradiation of the left side of the PTV in the BEV. In the composite delivery of many IMRT fields, this leads to a more or less uniform additional dose to the PTV.

We show that the maximum MLC opening can be confused with the dose errors due to the uncertainty of the MLC transmission *T*. These two effects are physically independent; however, we discuss them simultaneously in the context of dose errors because in the IMRT treatment‐planning system dosimetric parameters are treated as adjustable parameters so that the agreement between the plan and delivery is achieved by tweaking them. We derive a relationship between the fluence error ΔΦ and the uncertainty of the MLC transmission Δ*T*, which to our knowledge, has not been reported before. The log file analysis greatly facilitates discrimination of such competing effects in the dynamic MLC delivery. This understanding permits the user to optimize parameters to improve the quality of IMRT delivery when the undesirable features of the IMRT planning and record and verify systems cannot be completely removed.

## II. METHODS

### A. Dose rate‐dependence of leaf‐positional errors

In general, the magnitude of dynamic MLC delivery errors depends on dose rate *R* and total number of monitor units ${\text{MU}}_{\text{tot}}$, and it is constrained by the MLC tolerance TOL.^(5,9,10^) The reason for this is that MLC cannot move faster than the maximum physical speed $v_{\text{max}}$ and that its motion is controlled at a finite sampling rate (of about 50 ms). A more meaningful parameter than the physical MLC speed *v* (cm/s) is the MLC speed expressed in terms of fractional monitor units (mu):

(1) $$v(t)=\frac{\Delta x}{\Delta t}=\frac{\Delta x}{\Delta mu}\frac{R}{{MU}_{tot}}=v(mu)\frac{R}{{MU}_{tot}}\leq v_{\max,}$$

where Δ*x* is the increment in leaf position *x* and Δ*x*/Δmu is the slope of the leaf trajectory. In the above, we introduce a relative monitor unit fraction ${\text{mu}=\text{MU}/\text{MU}}_{\text{tot}}$ (“mu” is called “Index” in the MLC file; mu ∈ [0,1]). Thus the higher the *R*/MU_tot_ ratio, the smaller the upper bound on the velocity *v*(mu), and the larger the dose errors arising from the finite MLC controller sampling, and/or too high velocity *v*(mu) .

The total number of monitor units ${\text{MU}}_{\text{tot}}$ as well as the desired leaf trajectory *x*(mu) are determined by a treatment‐planning system (TPS) and therefore cannot be altered in the delivery for the patient. On the other hand, the dose rate may not be specified in the IMRT system (e.g., Novalis or Cadplan‐Helios) and can be altered; thus the user should select an optimal rate for the delivery. In some other IMRT systems (e.g., Eclipse‐Helios), the dose rate is specified at the MLC segmentation stage, but it is still possible to deliver the field with an altered (lower) dose rate than in the plan and expect, according to the manufacturer's design, no problems in the delivery. These altered dose rate schemes in IMRT delivery require a prudent verification. For instance, if the machine dose rate has a range R=160 MU/min to 800 MU/min, delivering with $R_{\min}=160\;\text{MU}/\text{min}$ is undesirable because of the long treatment time. At the other extreme, delivering with $R_{\max}=800\;\text{MU}/\text{min}$ may be too sensitive to leaf‐positional errors in general and may cause larger maxMLC errors in particular. In practice, one may need to compromise accuracy for time efficiency. The effect of the dose rate *R* on the leaf‐positional errors and dose errors can be determined by delivering IMRT fields for various *R* and analyzing the resulting DynLog™ (Varian) files, obtained for each plan during the QA process before the actual treatment.

### B. Quantification of errors due to the uncertainty in the MLC transmission

Generally, there are several MLC parameters in any IMRT software and hardware that may directly influence the dose calculation: MLC transmission *T* (typically from 1% to 2% of the open field doses), radiation field offset (RFO, the offset due to the penetration of radiation through the rounded leaf ends), and center mechanical offset (CMO, the artificial offset used to avoid mechanical collisions of the leaves during motion). A positive/negative offset (CMO+RFO>0) introduces a positive/negative fluence bias and was discussed earlier.^(9^) A positive/negative transmission error causes a positive/negative fluence bias as well.

For instance, for Varian MLCs, RFO is an important parameter due to the rounded leaf ends. In the Helios™ IMRT system, dosimetric parameters such as RFO, CMO, and transmission are treated as adjustable empirical parameters. In contrast, the Novalis MLC leaf ends are designed to be practically flat (RFO≈0 mm). The MLC can be very precisely set so that CMO≈0 mm. Thus, in the BrainLAB Novalis TPS configuration there is no such parameter as RFO or CMO. The only adjustable parameter is the transmission.

The importance of dose errors due to the uncertainty in the MLC transmission has been reported earlier.^(12)^ However, quantification of the transmission errors has not been presented so far. In the following, we adopt an approach based on the average fluence error.^(5,9)^

We assume that the fluence in the BEV(*x*, *y*) plane is expressed as

(2) $$\Phi (x,y)=(1-T)({MU}_{A}(x,y)-{MU}_{B}(x,y))+T\;{MU}_{tot},$$

where *T* is the MLC transmission, ${\text{MU}}_{A}$ and ${\text{MU}}_{B}$ are arrival times for the leaves belonging to leaf bank A and B, *x* is the leaf position along MLC motion, and *y* is the leaf number (y=1,2,…). Then the fluence error ΔΦ due to the uncertainty in the transmission Δ*T* is proportional to Δ*T*:

(3) $$\frac{{\left\langle \Delta \Phi \right\rangle }_{A_{PTV}}}{{\left\langle \Phi \right\rangle }_{A_{PTV}}}=\frac{\Delta T\left(1-\frac{ALPO}{w_{PTV}}\right)}{(1-T)\frac{ALPO}{w_{PTV}}+T}≅\Delta T\frac{1-\frac{ALPO}{w_{PTV}}}{\frac{ALPO}{w_{PTV}}}$$

In the above, $A_{\text{PTV}}$ is the effective area of PTV in the BEV, and $w_{\text{PTV}}$ is the effective width of PTV, and ALPO is the average leaf‐pair opening or the MLC gap.^(5,^, ^9)^ Also, $<\Phi {>}_{\text{APTV}}$ is the average fluence and $<\Delta \Phi {>}_{\text{APTV}}$ the average fluence error.

**Figure 3:**
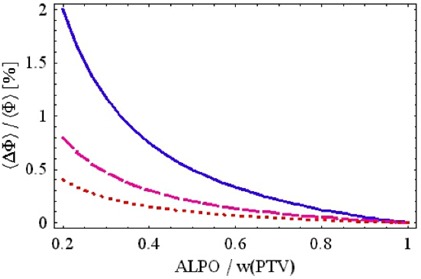
Relative average fluence error as a function of the $\text{ALPO}/w_{\text{PTV}}$ ratio for different values of ΔT=0.1% (dotted), 0.2% (dashed), 0.5% (solid), and T=1.5% according to Eq. (3). The fluence error would be negative for negative Δ*T* (a mirror image of the curves seen above).

From Eq. (3) it can be observed that the relative fluence error is proportional to the transmission error Δ*T* and approximately inversely proportional to the $\text{ALPO}/w_{\text{PTV}}$ ratio. The ratio $\text{ALPO}/w_{\text{PTV}}$ is typically 0.10 to 0.30, transmission T≈1.5%, and transmission error ΔT<0.5% of the open beam dose. Figure 3 shows that the fluence errors can be significant depending on the level of the uncertainty in ΔT(ΔT=0.1%,0.2%,0.5% and T=1.5% in the figure) and the $\text{ALPO}/w_{\text{PTV}}$ ratio.

This is an important insight because it indicates that a positive dose bias due to the maxMLC is typically confounded with a dose bias (negative or positive) due to Δ*T*. The maxMLC bias is not exactly uniform throughout the field (typically 1/3 to 2/3 of the field is overexposed on one side). However, the net result in a composite IMRT plan is a more or less uniform dose bias, especially if many IMRT fields are used. Thus, if in IMRT verification only dose is measured but no additional analysis of the actual leaf positions is performed, one may be under the impression that the value of the MLC transmission is incorrectly specified in the IMRT planning system.

### C. Numerical programs and experimental methods

We have written numerical programs to calculate ideal and delivered fluences based on MLC log files.^(9)^ The ideal and delivered fluences can be imported to the IMRT planning system for evaluation of the resulting doses. As an example, the Novalis (BrainLAB) TPS was used to generate IMRT plans, and MULTI‐ACCESS was used for IMRT delivery.

Experimental verification was performed with a diode array (MapCHECK™) at 100 source‐to‐detector distance with an inherent buildup of 2 cm in testing individual IMRT fields. The diode array was calibrated to measure dose in absolute units (cGy) according to the manufacturer's specifications. We also used films and an ionization chamber in a Rando phantom head and Lucite cylinder to test the overall effect of the maxMLC effects on a composite IMRT plan.

## III. RESULTS

### A. Experimental verifications of the maxMLC effect

**Figure 4:**
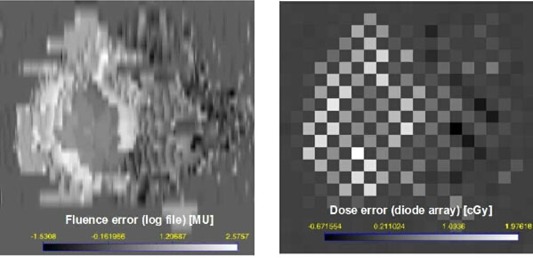
Absolute dose errors for a single IMRT field: calculated a log file fluence in MU (left) and measured diode array dose in cGy (right) (100 cGy is about equivalent to 100 MU). The bright regions indicate that the delivered dose was larger than the planned dose. Leaves were moving from left to right. The maxMLC overdose covers the left side of the PTV in the BEV. Local deviations from the ideal trajectory result in local fluctuations in dose and are seen as well. R=800 MU/min. The checkerboard pattern is due to the diode array geometry: diodes are 7 mm apart in a triangular array.

An example of the experimental verification of the maxMLC effect is presented in Fig. 4. The calculated fluence error (delivered minus ideal fluence) matches the measured dose error relative to the plan, both in the shape of the pattern and in absolute values (if the phantom scatter is taken into account). Local deviations from the ideal leaf trajectory result in local fluctuations in dose, which can be observed in the calculated and measured data. In the particular case of high dose rate R=800 MU/min, both the maxMLC errors and the local fluctuations are also large. The film irradiations showed the same patterns of extra dose but with a spatially higher resolution than the diode array measurements performed for the individual fields. We did not use the film as an absolute dosimeter but as a relative one. Ionization chamber measurements for the composite plans were in agreement with the diode array measurements performed for individual fields and summed for the purposes of the comparison (each diode representing a sum of contributions from individual fields with zero gantry, collimator, and couch angles). The composite diode signal showed an elevated level of doses in the left part of the IMRT fields, while the composite ionization chamber dose was elevated to a lesser degree because of the various beam configurations. The numerical values of the measured data varied for various patients but always showed the above features.

### B. Dose rate and total monitor unit dependence

**Figure 5:**
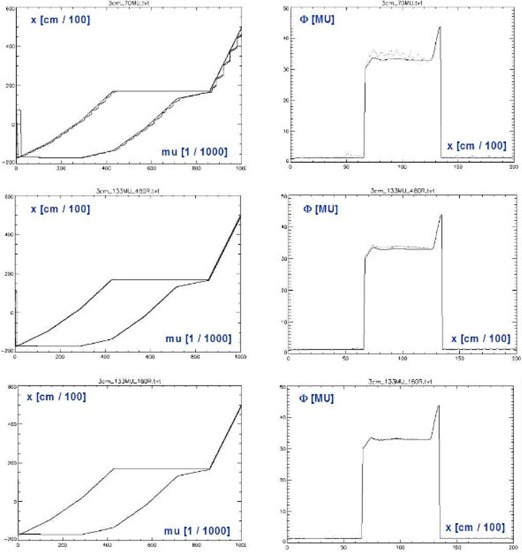
The same quantities as in but obtained for various combinations $\left(R[\text{MU}/\text{min}],{\text{MU}}_{\text{tot}}[\text{MU}])=(800,70),(480,133),(160,133\right)$. ${\text{MU}}_{\text{tot}}=133$ is the original number of monitor units calculated by the IMRT system. When the dose rate is decreased, the maximum opening artifact and the local wiggles in the MLC trajectory are decreased.

The maxMLC effect can be significantly altered by changing the dose rate *R* and/or the total number of monitor units ${\text{MU}}_{\text{tot}}$ per IMRT field. Figure 5 shows leaf‐positional and fluence errors corresponding to Fig. 2, but obtained for various pairs (*R* [MU/min], ${\text{MU}}_{\text{tot}}$ [MU]) = (800, 70), (480, 133), (160, 133). As expected, decreasing the number of monitor units increases the errors, and decreasing the dose rate decreases them.

**Figure 6:**
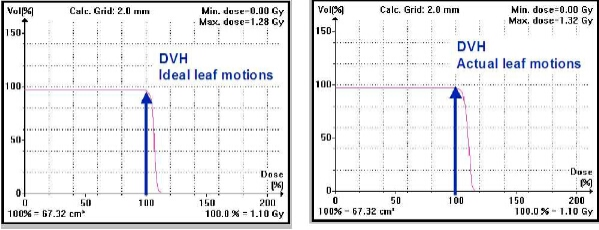
Dose‐volume histogram for an IMRT plan with imported ideal fluences (left) and imported delivered fluences (right). The larger dose on the right is due to the maxMLC effect. The fluences were calculated based on the MLC log files.

This underlines the importance of performing IMRT QA in exactly the same conditions as during the patient treatment, including the record and verify system (MULTI‐ACCESS in our case). The reason is that some of the dose errors do not scale with the delivery parameters in a simple fashion. Another insight is that decreasing the dose rate from 800 MU/min to 480 MU/min may not be sufficient to remove the maxMLC overdose completely. Decreasing the dose rate to 160 MU/min brings more dramatic improvement; however, it implies a fivefold increase in delivery time, which may not be acceptable. In practice, one may need to find a compromise between the decreased accuracy of delivery and the increased delivery time.

In addition to the maxMLC effect, the results in Fig. 4 show local fluctuations in leaf positions and the resulting dose errors (wiggles). The local fluctuations are smaller in magnitude, and they cancel each other partially in the composite plan. The effects related to random leaf‐positional fluctuations were reported before.^(5)^

### C. maxMLC effect for the composite plan

The maxMLC effect causes a uniform overdosage in a large portion of the PTV in the BEV. For a composite plan the total overdose may be slightly decreased, and it may become more uniform across the whole PTV. Figure 6 shows a dose‐volume histogram for two IMRT plans: one for an ideal delivery and the other for an actual delivery using R=800 MU/min. Effectively, PTV receives about 4% more dose due to maxMLC effect. This example is an extreme case. The dose rate of 800 MU/min is highly time‐efficient but poses a potential danger of elevated errors. For a typical IMRT plan delivered at R=800 MU/min, one could expect dose errors for individual fields to be 0% to 4%, with the effective dose error approximately 1% to 3% for a composite plan.

### D. Combined effect of the maxMLC and MLC transmission errors

Altering the value of MLC transmission *T* in the IMRT planning software can effectively raise or lower the dose for each field. For instance, assuming a larger value of transmission in the TPS than in reality will effectively decrease the monitor units per field calculated by the TPS. Decreased monitor units, in turn, lead to decreased physical dose. If the maxMLC occurs at the same time, the combined dose error for the composite plan is smaller than for each of the effects separately. This is due to partial cancelation of the biases of the opposite sign. We have confirmed this prediction by performing ionization chamber measurements for a composite IMRT plan and diode array measurements for individual IMRT fields. The degree of the cancelation depends on the specific dose rate *R* used in delivery, on the $\text{ALPO}/w_{\text{PTV}}$ ratio (Eq. (3)), and on Δ*T*. While the choice of *R* and *T* depends on the user, the performance of the MLC segmentation algorithm in the given TPS depends on the manufacturer of the IMRT system.

## IV. DISCUSSION

By analysis of the dMLC log file data, we have observed effects that may go unnoticed if only the resulting dose verification of IMRT is performed, for example, by point dose measurements or even by film measurements of the composite plan. The particular effect discussed is related to the presence of a maximum MLC opening as the first dMLC aperture. The maxMLC dose errors can confound the errors due to the uncertainty in MLC transmission Δ*T* (and vice versa). The log file analysis clearly shows not only the qualitative features of IMRT delivery errors but the quantitative ones as well. In order to assess the size of the dose errors it is suggested that the log file analysis be done for various dose rates to determine the optimal rate used in patient irradiation. Lower dose rates decrease both the local and maxMLC errors. However, one may compromise the treatment time at these lower rates. We suggest the dose rate of 480 MU/min or lower for the Novalis™ and MULTI‐ACCESS™ systems. With this dose rate one can expect a dose bias of the order of 1% to 3% on the average for a composite plan.

Ideally, the manufacturer of the IMRT system should take into account the limitations of dynamic MLC delivery and implement them in the MLC segmentation algorithm. However, at present there are no standard or generally agreed‐upon solutions. In the present version of the Novalis™ TPS (v5.3) and MULTI‐ACCESS™ record and verify (v6.10) systems, it is not possible to deliver IMRT without the maxMLC effect. Thus, a decision needs to be made for each patient if delivery parameters (e.g., dose rate) are too demanding. For instance, the dose rate of 480 MU/min may be too high; then the delivery of IMRT has to be performed at a lower dose rate. This can be readily determined by analysis of the dMLC log files obtained for various dose rates.

## V. CONCLUSIONS

We recommend avoiding the usage of the higher dose rates, for example, R=800 MU/min. The dose rate R=480 MU/min is a reasonable choice for most Novalis/IMPAC patients in terms of time efficiency of the treatment and the level of dose bias (up to +3%). For selected patients for which the dose bias reaches +5%, a lower dose rate of 160 MU/min can be used. With the current versions of the Novalis/IMPAC IMRT delivery, it is not possible to turn off the maximum MLC opening subfield. In the Novalis TPS configuration there is no such parameter as RFO or CMO; therefore, one cannot change them in there. The only adjustable parameter is the transmission. We have determined that the only way to partially correct for the maximum opening effect in Novalis/IMPAC delivery is by biasing the MLC transmission in the TPS configuration. However, we do not recommend this as a final solution. Rather, we suggest decreasing the dose rate during IMRT delivery. Nonetheless, biasing the transmission is an option, and one has to understand the potential interplay between the two: the maximum opening effect and an adjustable TPS parameter (transmission).
